# A short note on the use of irreducible representations for tilted octahedra in perovskites

**DOI:** 10.1107/S2052520624006668

**Published:** 2024-08-08

**Authors:** A. M. Glazer

**Affiliations:** a Clarendon Laboratory University of Oxford Parks Road Oxford OxfordshireOX1 3PU United Kingdom; b Department of Physics University of Warwick Coventry WarwickshireCV7 4AL United Kingdom; Adam Mickiewicz University, Poland

**Keywords:** perovskite, irreducible representations, tilt, octahedra

## Abstract

It is pointed out that many authors are unaware that the particular choice of unit-cell origin determines the irreducible representations to which octahedral tilts in perovskites belong. Furthermore, a recommendation is made that the preferred option is with the origin at the *B*-cation site rather than that of the *A* site.

## Abstract 2

Comment on the use of irreducible representations to denote tilting of octahedra in perovskites.

## 1. Introduction

The crystal structure of perovskite, formula *ABX*_3_ (*A*, *B* cations, *X* anions), is capable of a considerable number of structural variations. Its *aristotype* structure (Megaw, 1973 ▸) is cubic in space group 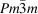. In this space group, there are two equivalent choices for the origin of the unit cell (Table 1 ▸). Option 1 places the *B* cation at the origin of the unit cell, while option 2 places the *A* cation at the unit-cell origin. Either choice places the *B* cation at the centres of the *X* octahedra and the *A* cation within the space between the octahedra. As is well known (Megaw, 1973 ▸), this basic structure type is capable of many structural variations involving cation displacements, octahedral tilting, and octahedral distortions. These variations are generally slight and make it possible to consider the different structures (*hettotypes*) with respect to tilting about the three nearly perpendicular pseudocubic axes.

The focus of this note is on octahedral tilting. The first crystal structure determination in which tilted octahedra in perovskites were found seems to have been by Náray-Szabó (1943 ▸) for CaTiO_3_.^1^ Glazer (1972 ▸) and independently Aleksandrov (1976 ▸) introduced a notation to describe the tilting of octahedra in perovskite crystal structures. Small tilts of the octahedra about each of the pseudocubic axes, *a*, *b* and *c*, were considered with in-phase (+) and antiphase (−) tilting *about* each axis in turn. This gave rise to ten distinct tilt patterns. On inclusion of the equalities or otherwise of the tilt angles, 23 tilt structure types were found, accommodated in 15 distinct space groups (Howard & Stokes, 1998 ▸). The result was a convenient notation that is now internationally accepted.

An alternative way of addressing the two types of tilting is by using irreducible representations (irreps), which are used to describe so-called distortion modes. This is particularly useful when considering phase transitions in perovskites where one thinks, in the case of tilts, of the condensation of phonon modes with wavevectors ending at the Brillouin zone boundaries. With respect to the cubic Brillouin zone, (+) tilts can be thought of as arising from phonons with wavevectors at the *M* points (½, ½, 0 *etc*.) and (−) tilts at the *R* points (½, ½, ½ *etc*.). However, it appears not to be realized by many authors of publications on perovskites (and for that matter, also for other crystal structures, such as layer perovskites) that the actual irreps for wavevectors at the Brillouin zone boundaries depend on the choice of origin chosen to describe the particular crystal structure. The two choices which are related by an operation of the Euclidean normalizer are summarized in Table 2 ▸. Often, authors use the irrep symbols for option 1 as standard, apparently unaware that it is necessary to specify the unit-cell origin. Some papers use the irreps listed for option 1, even though the crystal structure is described using option 2! Sometimes, the cell choice is not given at all. This is clearly misleading and adds to the confusion over the irreps found in the literature. It is worth noting that the often-used software *ISODISTORT* (Campbell *et al.*, 2006 ▸) employs option 1 for its default sample perovskite structure, and this leads to the usual specification 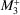 for in-phase tilts and 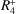 for antiphase tilts, the most commonly used choice in the literature [see, for example, Howard & Stokes (1998 ▸) and Bechtel & Van der Ven (2018 ▸)]. On the other hand, the program *AMPLIMODES* (Orobengoa *et al.*, 2009 ▸; Perez-Mato *et al.*, 2010 ▸) in the Bilbao Crystallographic Server (Aroyo *et al.*, 2006 ▸) uses option 2 for its example structure, thus leading to 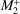 and 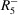.

**Figure 1:**
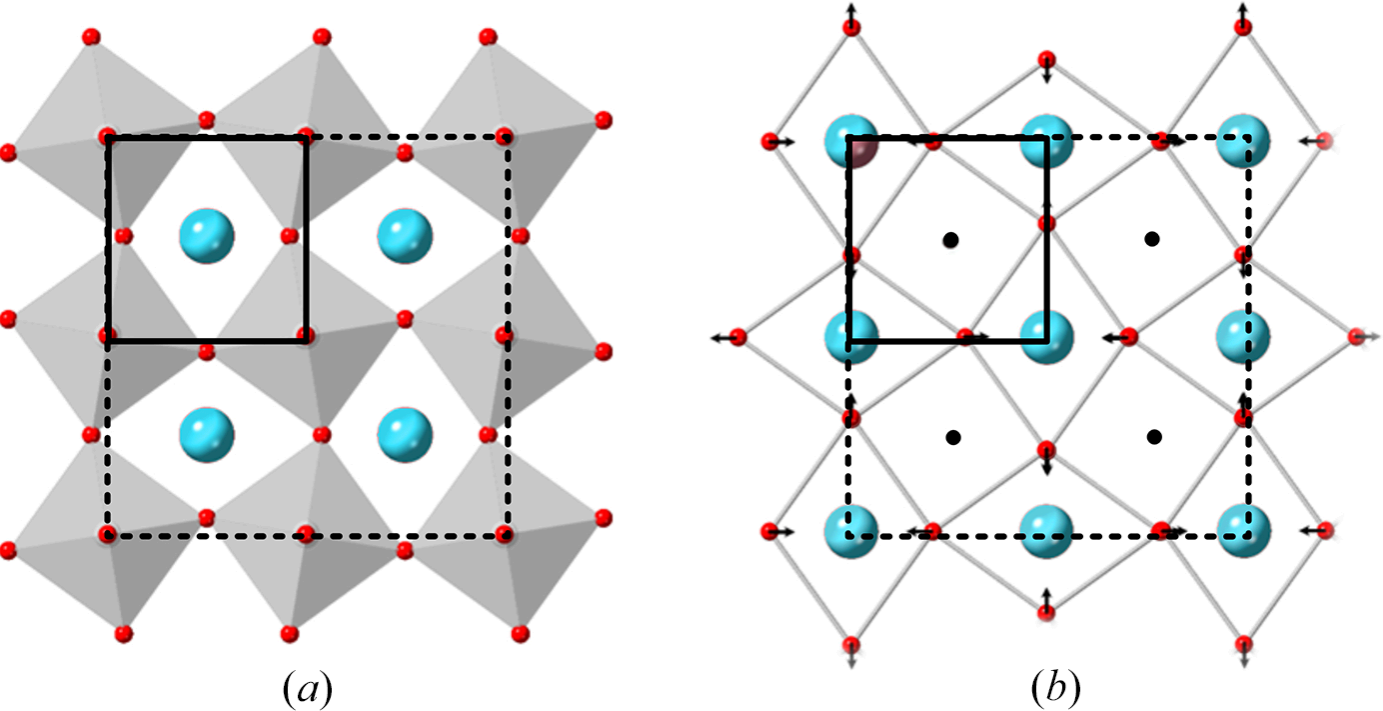
(*a*) The effect of tilting of the octahedra about the axis perpendicular to the octahedra plane. (*b*) The pattern of the *X* anion shifts with respect to the *A* cations after tilting. *X* anions are in red, *A* cations are in blue. The *B* cation (black) is located within the octahedra. The pseudocubic unit cell is marked in each case by continuous black lines and the doubled supercell by dashed lines.

This raises the question of whether a particular option can be recommended. Of course, in principle, both are correct and equally valid, provided that the unit-cell origin is stated explicitly. However, I would argue that option 1 should be the normal convention. Consider Fig. 1 ▸(*a*), in which a layer of the octahedra is shown following tilting about the axis perpendicular to the projection plane. As is now well known, tilting of one octahedron about this axis affects all the other octahedra within the projection plane, thus causing a doubling of the unit-cell edges to form a superstructure (in Megaw’s 1973 terminology, this structure is a hettotype). When drawn in this way, it is evident that the best way to view this structure is with respect to the six-coordination polyhedron about the *B* cation, thus leading to the usual description of the perovskite structure as consisting of corner-linked octahedra.

However, an alternative way of thinking about the structure [Fig. 1 ▸(*b*)] is with respect to the 12-coordination polyhedron (cuboctahedron) for the *A* cation (or virtual *A* cation site in cases where the actual *A* cation is missing, such as in WO_3_ and ReO_3_) (Woodward, 1997 ▸). In this picture, the *X* anions are seen to move towards and away from the *A* site. This forms a diamond-shaped pattern in projection, with neighbouring diamonds alternating in orientation, thus doubling the unit-cell edges. The linking of these coordination polyhedra for the hettotypes provides a different way of thinking from that of corner-linked octahedra, even though it is possible to describe the perovskite structures in this way. This is not done in practice because it is much more challenging to visualize, especially when considering tilts about all the pseudocubic axes.

In conclusion, authors writing papers on octahedral tilting in perovskites need to be aware that the correct use of irreps depends on the choice of the origin of the unit cell, with option 1 being preferred in order to be consistent with the majority of publications. If the exact irrep is unimportant for the discussion in a given publication, then it is better not to specify an irrep at all but merely to use the phrases ‘in-phase’ or ‘antiphase’ tilts. Or else, an author could simply describe the tilts associated with the *R* or *M* point of the cubic Brillouin zone as appropriate without specifying the actual irrep label.

## Figures and Tables

**Table 1:** Two origin choices for aristotype unit cell in 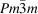

	Option 1		Option 2	
*A*	1*b*	½, ½, ½	1*a*	0, 0, 0
*B*	1*a*	0, 0, 0	1*b*	½, ½, ½
*X*	3*d*	½, 0, 0	3*c*	0, ½, ½

**Table 2:** Standard symbols for irreps for in-phase and antiphase octahedral tilts

	Option 1	Option 2
+ tilting	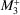	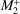
− tilting	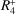	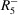

## Footnotes

1 Nara-Szabo gave the space group as *P*2_1_/*m* and found a complex three-dimensional set of octahedral tilts. Unfortunately, his publication gives little information as to how this impressive feat was achieved as long ago as 1943. Despite this, we now know that the correct room-temperature space group is *Pbnm*.
